# Relationship between carotid-femoral pulse wave velocity and prognosis in maintenance hemodialysis patients

**DOI:** 10.1097/MD.0000000000039099

**Published:** 2024-08-09

**Authors:** Yifan Zhu, Juan Li, Min Ding, Fengping Qiu, Qi Zhao, Hulin Lu, Lingyan Ren, Zhanqin Shi

**Affiliations:** a Department of Nephrology, The First People’s Hospital of Huzhou, Huzhou, Zhejiang Province, China; b Tongxiang Chinese Medicine Hospital, Tongxiang, Zhejiang Province, China.

**Keywords:** arterial stiffness, carotid-femoral pulse wave velocity, maintenance hemodialysis, prognosis

## Abstract

Carotid-femoral pulse wave velocity (Cf-PWV) can well predict the prognosis of the general population. However, whether Cf-PWV can be used as a prognostic indicator in maintenance hemodialysis (MHD) patients remains mysterious. The present study endeavored to explore the prognostic value of Cf-PWV among the MHD population. Patients who received MHD and underwent Cf-PWV examination at the hemodialysis center of Zhejiang Provincial People’s Hospital between March 1, 2017 and October 15, 2019 were enrolled. Relevant clinical data were collected from these patients, who were subsequently followed up for a minimum of 1 year. During the follow-up period, the occurrence of all-cause death was recorded as a prognostic indicator. Based on the predetermined inclusion and exclusion criteria 178 patients were included in the final analysis. These patients were categorized into 2 groups based on Cf-PWV values: group 1 (Cf-PWV < 13.8 m/s), and group 2 (Cf-PWV ≥ 13.8 m/s). Thirty-four patients succumbed to their conditions within a median follow-up period of 23.3 months. Kaplan–Meier survival analysis revealed that the median survival time of group 2 was significantly shorter than group 1 (log-rank test, *χ*^2^ = 12.413, *P* < .001). After adjusting for various factors, including age, cardiovascular disease, peripheral arterial diastolic pressure, central arterial diastolic pressure, albumin, blood urea nitrogen, serum creatinine, left ventricular ejection fraction, 25 hydroxyvitamin D3, C-reactive protein and serum phosphorus, it was found that Cf-PWV ≥ 13.8m/s was an independent risk factor for all-cause mortality in MHD patients (relative risk = 3.04, 95% confidence interval [CI] = 1.22–7.57; *P* = .017). A high level of Cf-PWV (≥13.8 m/s) is an independent risk factor for all-cause death in MHD patients.

## 1. Introduction

Despite the ongoing advancements in hemodialysis technology and equipment, the mortality rate among maintenance hemodialysis (MHD) patients remains considerably high.^[1]^ Among the various factors contributing to mortality, cardiovascular disease (CVD) remains the predominant cause of death in the MHD population.^[2,3]^ Furthermore, research has established arteriosclerosis, encompassing arterial stiffness and atherosclerosis, as an autonomous risk factor for CVDs in individuals with diabetes mellitus or hypertension. Moreover, it is noteworthy that large artery stiffness is closely associated with an increased risk of cardiovascular and all-cause mortality (ACM). This risk may surpass the predictive ability of conventional risk factors, such as hypertension and diabetes.^[4,5]^ Various methods have been employed to assess arteriosclerosis, specifically pulse wave conduction velocity. These methods include digital subtraction angiography, intravenous ultrasound, and indirect pulse wave velocity (PWV) measurements encompassing noninvasive imaging, carotid-femoral pulse wave velocity (Cf-PWV), brachial-ankle PWV, and finger-toe PWV.^[6]^ PWV refers to the rate at which the arterial pulse – resulting from cardiac ejection – propagates along the arterial wall. This parameter serves as an indicator of arterial elasticity and arteriosclerosis risk. A higher PWV value corresponds to a greater degree of arteriosclerosis. It can also help us identify subclinical arteriosclerosis.^[7]^ The assessment of Cf-PWV offers several advantages, including noninvasiveness, strong operability, high accuracy, and good repeatability. Consequently, it serves as the optimal alternative for measuring arterial stiffness. Moreover, Cf-PWV is currently regarded as the clinical gold standard for assessing arterial stiffness.^[8]^ Cf-PWV has demonstrated promising prognostic capabilities in the general population and non-dialysis patients with chronic kidney disease.^[9–12]^ However, its ability to accurately predict the prognosis of MHD patients remains controversial. Therefore, the current study sought to explore the relationship between Cf-PWV and prognosis in an MHD population.

## 2. Methods

### 2.1. Participants

A total of 178 patients who underwent MHD at the hemodialysis center of Zhejiang Provincial People’s Hospital between March 1, 2017 and October 15, 2019 were recruited. All patients underwent an examination of PWV. The inclusion criteria were as follows: patients who had been on MHD for over 3 months, patients who had undergone MHD 3 times a week for a minimum of 4 hours each time, and patients aged at least 18 years old. The exclusion criteria were as follows: failure to meet the predetermined standard in the measured score (<80 points), incomplete data, and changing dialysis mode or kidney transplant during the specified period.

### 2.2. Data collection

Demographic data collected included gender, age, height, weight, smoking status, dialysis age, vascular access, and complications (such as hypertension, diabetes, CVD, and tumor history). Additionally, laboratory indicators of patients within the preceding 30 days were collected, including hemoglobin, white blood cells, red blood cells, platelets, C-reactive protein, serum albumin (ALB), blood urea nitrogen, serum creatinine, blood uric acid, total cholesterol, triglycerides, low-density lipoprotein cholesterol, N-terminal osteocalcin, type 1 procollagen amino-terminal peptide, 25 hydroxyvitamin D3 (25(OH)-VD3), and other relevant parameters. Furthermore, the dimensions of cardiac structure and function, such as aortic diameter, left atrial diameter, right ventricular diameter, left ventricular ejection fraction (LVEF), left ventricular end-diastolic diameter, left ventricular end-systolic diameter, ventricular septal thickness, and left ventricular posterior wall thickness, were assessed using color Doppler echocardiography (Mindray, DC-35 Pro, Shenzhen, China). Additionally, the left ventricular mass was determined using an internationally recognized formula.

### 2.3. Cf-PWV measurement

The measurement of Cf-PWV was acquired in the supine position following a 10-minute period of rest. This was accomplished using the SphygmoCor^®^ CvMS device (AtCor, Australia, 2013) and accompanying software. The tester affixed a cuff around the patient’s femoral artery to capture the femoral waveform, while the Millar tonometer pressure sensor was placed at the patient’s carotid artery to capture the carotid waveform. Both waveforms were continuously recorded for 10 to 20 seconds and documented on the electrocardiogram. The conduction velocity of the Cf-PWV was calculated using a computer, employing the following formula: Cf-PWV = distance (*L*)/propagation time (△*T* − Cf) (m/s). The distance refers to the length between the carotid artery and the femoral artery, while the propagation time denotes the time discrepancy between the 2 waveforms (△*T* − Cf).

### 2.4. Follow-up

Follow-up methods included review of the electronic medical record system via the telephone. Death events, time of death and cause of death were recorded during the follow-up period.

### 2.5. Statistical analysis

All statistical analyses were conducted using SPSS version 25.0, GraphPad Prism 8 and R version 3.5.3. Normally distributed quantitative variables were presented as means and standard deviations, whereas non-normally distributed variables were expressed as medians and interquartile ranges. Categorical data were summarized using ratios and percentages. One-way analysis of variance was employed to compare normally distributed data among 3 groups, whereas the Kruskal–Wallis *H* test, a multiple independent sample rank sum test, was utilized for non-normally distributed data. Survival was estimated using the Kaplan–Meier product-limit method and compared using the Mantel (log-rank) test. The multivariate Cox proportional hazards regression model was employed to examine the independent association between parameters and the outcomes. A 2-sided *P* value of <.05 was considered statistically significant.

## 3. Result

### 3.1. Baseline characteristics

Overall, 178 patients were included in the final analysis, of which 62 (34.8%) were female and 52 (29.5%) were smokers. The mean age was 61 ± 16 years. Sixty-four patients (36.0%) had primary glomerulonephritis, 59 patients (33.1%) had diabetes nephropathy, 18 patients (10.1%) had hypertensive renal damage, 9 patients (5.1%) had polycystic kidney disease, 8 patients (4.5%) had tubulointerstitial kidney disease, and 20 patients (11.2%) had other unknown causes. In terms of complications, 52 patients (29.2%) had diabetes, 139 patients (78.1%) had hypertension, and 62 patients (34.8%) had cardiovascular disease.

Thirty-four all-cause deaths were observed within a median follow-up period of 23.3 months. Among them, 7 died from heart-related issues, 5 from strokes, 5 from cancer, 5 from infections, 2 from other reasons, and 10 from unknown causes. Table 1 compares baseline data between death and survival groups. The 2 groups exhibited significant differences in terms of age, Cf-PWV, cardiovascular disease, peripheral arterial diastolic pressure, central arterial diastolic pressure, albumin, blood urea nitrogen, serum creatinine, LVEF, 25 hydroxyvitamin D3, C-reactive protein, and serum phosphorus.

**Table 1:** Comparison of baseline characteristics between survival group and death group.

Variables	Overall	Survival group (n = 144)	Death group (n = 34)	*t/Z*	*P*
Age (yr)	64 (49,73)	60 (46, 68)	75 (69, 82)	−5.765	<.001
Cf-PWV	10.1 (8.7, 14.0)	10.7 (8.6, 13.6)	13.7 (9.3, 16.1)	−2.374	.018
Female (%)	62 (34.8)	51 (35)	11 (32)	−0.336	.737
Duration of dialysis (mo)	24.5 (8.2, 73.4)	25.6 (8.3, 76.9)	24.5 (7.5, 60.9)	−0.540	.589
BMI (kg/m^2^)	22.5 ± 3.3	22.4 ± 3.4	22.1 ± 3.5	0.516	.606
History of smoking, n (%)	52 (29.2)	44 (31)	8 (24)	−0.808	.419
Hypertension, n (%)	139 (78.1)	110 (76)	29 (85)	−1.126	.260
Diabetes, n (%)	52 (29.2)	41 (28)	11 (32)	−0.446	.655
History of CVD, n (%)	62 (34.8)	44 (31)	18 (53)	−2.457	.014
P_SP (mm Hg)	147.5 ± 19.0	148.8 ± 19.5	148.8 ± 20.7	0.012	.990
P_DP (mm Hg)	80 (74, 90)	83 (75, 90)	79.5 (69, 89)	−2.125	.031
C_SP (mm Hg)	133.8 ± 18.8	135.8 ± 18.9	132.7 ± 20.0	0.847	.398
C_DP (mm Hg)	82.2 ± 14.4	84.8 ± 14.0	79.1 ± 12.7	2.170	.031
C_AIX_HR75	29 (20, 35)	29 (20, 35)	27.5 (20, 34)	−0.505	.613
ALB (g/L)	36.3 (32.6, 38.5)	37.0 (33.8, 39)	33.1 (31, 36.1)	−3.765	<.001
BUN (mmol/L)	18.3 (14.4, 23.4)	19.0 (15.0, 23.8)	16.2 (11.9, 20.6)	−2.792	.005
UA (mmol/L)	373.3 ± 122	373.9 ± 121.2	355.0 ± 135.6.2	0.799	.425
Na^+^ (mmol/L)	140.0 ± 2.9	140.0 ± 2.8	139.8 ± 3.1	0.397	.692
SCR (μmol/L)	635 (484, 879)	675 (523, 908)	513 (412, 814)	−3.136	.002
K^+^ (mmol/L)	4.70 ± 0.66	4.72 ± 0.67	4.78 ± 0.81	−0.459	.647
Ca^2+^ (mmol/L)	2.24 ± 0.21	2.22 ± 0.24	2.23 ± 0.22	−0.222	.824
P (mmol/L)	1.85 ± 0.59	1.87 ± 0.62	1.58 ± 0.59	2.487	.014
TC (mmol/L)	4.02 (3.26, 4.88)	4.03 (3.35, 4.89)	3.77 (3.12, 4.62)	−0.973	.330
TG (mmol/L)	1.28 (0.93, 1.74)	1.30 (0.99, 1.74)	1.08 (0.80, 1.45)	−1.791	.073
LDL-C (mmol/L)	2.22 (1.69, 2.80)	2.22 (1.72, 2.86)	2.11 (1.48, 2.58)	−1.019	.308
Hb (g/L)	100.7 ± 20.5	100.8 ± 21.8	97.4 ± 20.6	0.815	.416
WBC (×10^9^/L)	5.48 (4.48, 7.11)	5.44 (4.52, 6.94)	5.75 (4.31, 8.81)	−0.796	.426
PLT (×10^9^/L)	171 (121, 212)	176 (127, 212)	158 (117, 198)	−0.551	.581
CRP (μg/L)	2.7 (1.3, 14.2)	2.4 (1.3, 10.1)	11.7 (2.0, 43.9)	−3.153	.002
25(OH)-VD3 (IU)	18.5 (13.7, 24.9)	18.5 (14.4, 26.5)	15.2 (8.8, 19.8)	−2.752	.006
i-PTH (pg/mL)	239 (141, 479)	239 (145, 449)	201 (99, 421)	−1.405	.160
LVEF (%)	62 (57, 65)	62 (60, 65)	58 (52, 63)	−2.545	.011
LVMI (g/m^2^)	119 (103, 140)	119 (103, 138)	119 (98, 147)	−0.293	.770
Fe (μmol/L)	233 (80,532)	233 (73,501)	272 (123, 745)	−1.151	.250
Pre-SP (mm Hg)	150.6 ± 22.0	151.4 ± 21.8	147.0 ± 22.7	1.051	.295

25(OH)-VD3 = 25 hydroxyvitamin D3, ALB = albumin, BUN = blood urea nitrogen, Ca^2+^ = calcium, C_AIX_HR75 = augmentation index adjusted for a 75 bpm heart rate, C_DP = central arterial diastolic pressure, C_SP = central arterial systolic pressure, Cf-PWV = carotid femoral pulse wave velocity, CRP = C-reactive protein, Fe = serum iron, Hb = hemoglobin, i-PTH = intact parathyroid hormone, K^+^ = potassium, LDL_C = low density lipoprotein cholesterol, LVEF = left ventricular ejection fraction, LVMI = left ventricular mass index, Na^+^ = serum sodium, P = serum phosphorus, P_DP = peripheral arterial diastolic pressure, P_SP = peripheral arterial diastolic pressure, PLT = platelet, Pre-SP = pre-dialysis systolic blood pressure, SCR = serum creatinine, TG = triglycerides, UA = serum uric acid, WBC = white blood cell.

### 3.2. Survival analysis

**Figure 1.**
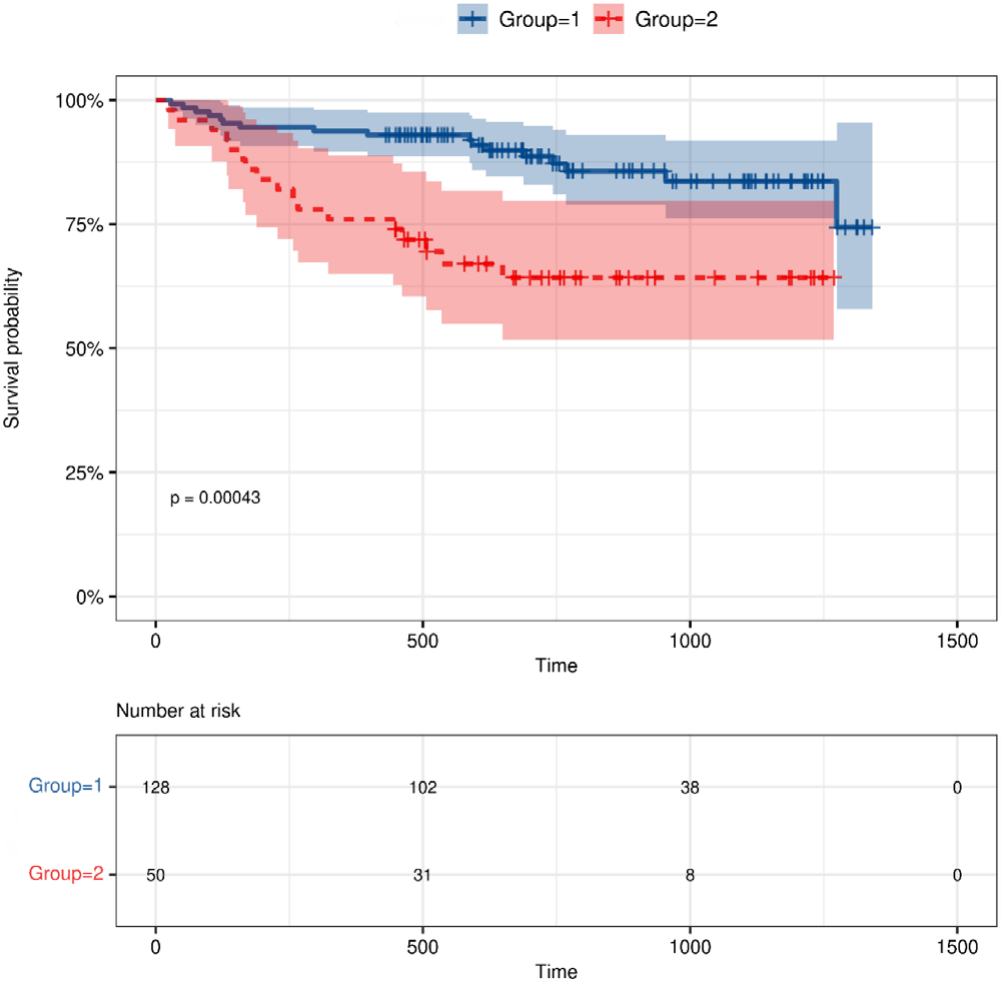
Unadjusted Kaplan–Meier cumulative-event curves for all-cause mortality. The median survival time of group 2 was significantly shorter than group 1 (log-rank test, *χ*^2^ = 12.413, *P* < .001), group 1, Cf-PWV < 13.8 m/s; group 2, Cf-PWV ≥ 13.8 m/s. Cf-PWV = carotid-femoral pulse wave velocity.

We use the Youden index in ROC curve to calculate the Cf-PWV cutoff (Cf-PWV ≥ 13.8m/s) for ACM. Then, these patients were categorized into 2 groups based on Cf-PWV values: group 1 (Cf-PWV < 13.8 m/s), and group 2 (Cf-PWV ≥ 13.8 m/s). Survival rates were compared between the 2 groups using Kaplan–Meier survival curves. The results revealed that the median survival time of group 2 was significantly shorter than group 1 (log-rank test, *χ*^2^ = 12.413, *P* < .001; Fig. 1).

### 3.3. Cox proportional hazards model analysis

**Figure 2.**
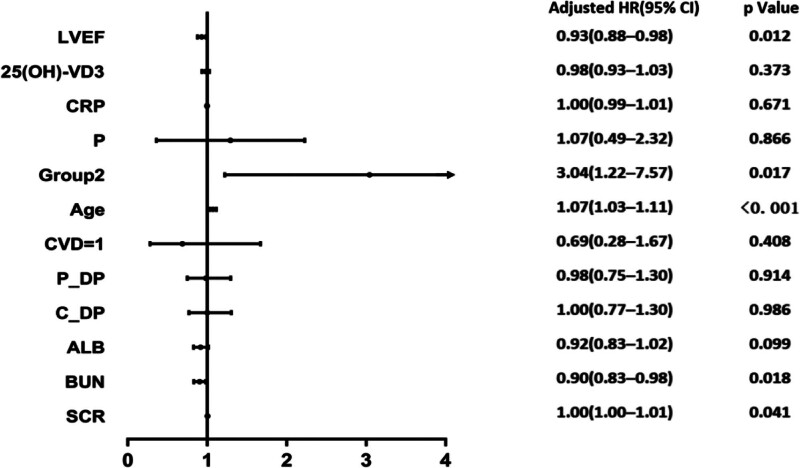
Cox regression model between carotid-femoral pulse wave velocity and all-cause mortality. After adjusting for age, cardiovascular disease, peripheral arterial diastolic pressure, central arterial diastolic pressure, albumin, blood urea nitrogen, serum creatinine, left ventricular ejection fraction, 25 hydroxyvitamin D3, C-reactive protein and serum phosphorus, it was found that Cf-PWV ≥ 13.8 m/s was an independent risk factor for all-cause mortality in MHD patients (relative risk [RR] = 3.04, 95% confidence interval [CI] = 1.22−7.57; *P* = .017). CVD=1, with history of CVD, is categorical variable. Group2, Cf-PWV ≥ 13.8 m/s, is categorical variable. Peripheral arterial diastolic pressure, central arterial diastolic pressure, albumin, blood urea nitrogen, serum creatinine, left ventricular ejection fraction, 25 hydroxyvitamin D3, C-reactive protein and serum phosphorus are continuous variables. 25(OH)-VD3 = 25 hydroxyvitamin D3, ALB = albumin, BUN = blood urea nitrogen, C_DP = central arterial diastolic pressure, CRP C-reactive protein, CVD = cardiovascular disease, LVEF = left ventricular ejection fraction, P = serum phosphorus, P_DP = peripheral arterial diastolic pressure, SCR = serum creatinine.

All variables showing *P* value < .05 in univariable analysis were included in a multivariable Cox regression model. After adjustments for confounding factors, it was found that Cf-PWV ≥ 13.8 m/s was an independent risk factor for ACM in MHD patients (relative risk = 3.04, 95% CI = 1.22–7.57; *P* = .017; Fig. 2).

## 4. Discussion

The mortality rate among patients with end-stage renal disease (ESRD) was found to be 18.3 times greater than that of the general population.^[13]^ CVDs have been identified as the leading cause of mortality in MHD population worldwide. The US Renal Data System 2019 Annual Data Report on kidney disease revealed that more than half (55.2%) of deaths with a known cause were attributed to CVDs.^[14]^ In recent years, numerous studies have demonstrated that Cf-PWV can effectively predict cardiovascular events (CVEs) and cardiovascular mortality, regardless of age and blood pressure.^[15–17]^ It is also believed that the predictive capacity of Cf-PWV surpasses that of established conventional cardiovascular risk factors such as blood pressure and smoking.^[18]^ However, whether Cf-PWV can effectively forecast the prognosis of MHD patients remains controversial. Verbeke et al^[19]^ conducted a 2-year longitudinal study involving 1084 MHD patients from 47 European dialysis centers. They found that PWV can serve as an independent prognostic indicator for both ACM and non-fatal CVEs. After adjusting for age, diabetes, serum ALB, and abdominal aortic calcification score, the risk of ACM increased by 0.15 (95% CI = 1.085−1.228, *P* < .001) for every 1 m/s increment in PWV. Blacher et al^[20]^ reached a similar conclusion that PWV holds potential as a prognostic indicator for individuals undergoing MHD. Conversely, Otsuka et al^[21]^ found that PWV did not exhibit independent predictive capabilities for major adverse cardiovascular events when accounting for associated risk factors. Their study encompassed 104 MHD patients, with major adverse cardiovascular events serving as prognostic indicators, and a median follow-up duration of 3.6 years. A recent retrospective study by Vongsanim and Davenport^[22]^ explored the relationship between PWV and the prognosis of MHD patients and revealed that while dialysis patients with elevated PWV exhibited a markedly increased risk of ACM, PWV ceased to be an independent risk factor for ACM after adjustments for age and complications.

The present study further supports the findings of Verbeke et al^[19]^ and Blacher et al^[20]^ that PWV can serve as a prognostic indicator for MHD patients. Although the initial study by Blacher et al^[20]^ has a limited sample size, it provides valuable guidance for subsequent research. Contrarily, Verbeke et al^[19]^ employed a more robust research design with a significantly larger sample size of 1084 cases. Their study not only demonstrated the predictive capability of Cf-PWV in relation to the occurrence and mortality of CVEs in patients but also emphasized the significance of the arterial calcification score. Vongsanim and Davenport^[22]^ initially demonstrated through univariate analysis that there was a significant association between PWV and heightened mortality. Nevertheless, subsequent adjustments for age, sex, and the Charlson Comorbidity Index revealed that PWV did not retain its status as an independent risk factor for mortality. Instead, only age and the Charlson Comorbidity Index were strongly correlated with mortality. Although notable similarities exist between our study and Vongsanim and Davenport’s study, the reported outcomes exhibited significant variation. Vongsanim and Davenport’s study divided patients into 2 groups based on whether PWV ≥ 10 m/s. Their findings indicated that PWV ≥ 10 m/s was not an independent risk factor for ACM. According to literature review, the 2007 European Guidelines for Hypertension Management initially suggested a cutoff value of 12 m/s to identify subclinical organ damage, which was subsequently revised to 10 m/s in 2015. However, it is noteworthy that this cutoff value was established based on findings from a hypertensive population rather than MHD population. Our study coupled with several previous studies has consistently demonstrated significantly elevated levels of Cf-PWV in MHD patients compared with the general population. Thus, it appears inappropriate to utilize the 10 m/s cutoff value when examining the prognosis of MHD patients. In our study, we use the Youden index in ROC curve to calculate the Cf-PWV cutoff for ACM. Then use Cox analysis to confirm that Cf-PWV ≥ 13.8 m/s was an independent risk factor for ACM in MHD patients.

Previous research on Cf-PWV in MHD patients has predominantly focused on European and American populations. However, there is a noticeable scarcity of studies on other ethnic populations, with existing studies limited to establishing correlations. The present study assessed the association between Cf-PWV and prognosis in a Chinese population, representing a novel contribution to the field. Nevertheless, this study has several limitations. As a single-center retrospective study, there is a possibility of selection and measurement outcome biases. Besides, our study findings lack external validity. Furthermore, this study has an insufficient follow-up period and sample size. As a result, there is a possibility of overestimating the survival time of MHD patients, which potentially compromises the accuracy of our conclusions. Hence, large-scale multicenter studies with a longer follow-up period are warranted. Additionally, although the inclusion of cardiovascular death data was a crucial component of our follow-up data, the lack of in-hospital deaths among the majority of the 34 patients observed in our study poses a significant challenge in ascertaining the precise cause of their death. Consequently, the association between Cf-PWV and cardiovascular mortality in MHD patients remains indeterminate due to the inherent difficulty in obtaining precise data on the underlying causes of death in this patient population.

## 5. Conclusion

A high level of Cf-PWV (≥13.8 m/s) is an independent risk factor for all-cause death in MHD patients.

## Acknowledgments

The authors are solely responsible for the contents of this paper.

## Author contributions

**Conceptualization:** Yifan Zhu, Juan Li.

**Data curation:** Juan Li, Qi Zhao.

**Formal analysis:** Min Ding, Fengping Qiu, Hulin Lu.

**Methodology:** Hulin Lu.

**Software:** Fengping Qiu.

**Writing – original draft:** Yifan Zhu.

**Writing – review & editing:** Lingyan Ren, Zhanqin Shi.
